# Rainstorm effects on the biocontrol efficacy of the decay fungus *Chondrostereum purpureum* against birch sprouting in boreal forests

**DOI:** 10.1007/s00253-020-10574-3

**Published:** 2020-04-02

**Authors:** Leena Hamberg, Veli-Matti Saarinen, Markku Rantala, Jarkko Hantula, Pekka Seiskari, Timo Saksa

**Affiliations:** 1 Natural Resources Institute Finland, Latokartanonkaari 9, FI-00790 Helsinki, Finland; 2 Natural Resources Institute Finland (Luke), Juntintie 154, FI-77600 Suonenjoki, Finland; 3 Verdera Ltd, P.O. Box 5, Kurjenkellontie 5 B, FI-02270 Espoo, Finland

**Keywords:** Sprout control, Formulation, adjuvant, stump mortality, *Betula*

## Abstract

**Abstract:**

In forest regeneration areas, alongside roads and railways, under electric power lines and above gas pipe lines, there is a need for regular sprout control. A biocontrol method against broadleaved sprouting with formulations including the decay fungus *Chondrostereum purpureum* (Pers. Ex Fr.) Pouzar has been shown to be effective. Yet, heavy rain during spreading of this fungal inoculum on freshly cut stumps may affect the efficacy of the treatment, i.e., stump mortality during the following years. Thus, we performed an experiment where freshly cut birch stump surfaces (*Betula pendula* Roth and *Betula pubescens* Ehrh.) were treated with fungal inoculum under heavy irrigation and without it. Furthermore, two different adjuvants which aimed to fix the fungal inoculum to freshly cut stumps during irrigation and to protect against solar radiation were tested. Our results revealed that the artificial rainstorm treatment caused a delay in the efficacy of *C. purpureum*, but after three growing seasons, there was no significant difference in the mortality of birch stumps treated under irrigation or without it (stump mortalities 74 and 86%, respectively). Adjuvants did not improve the efficacy in stumps treated under irrigation nor in those treated without irrigation.

**Key Points:**

*• Heavy rain delayed the sprout control efficacy of a fungus Chondrostereum purpureum.*

*• Final efficacy of formulations was the same in wet and dry conditions.*

*• No additional adjuvants are needed to improve formulations.*

## Introduction

In Finland, ca. 58 million euros are used annually for young stand management operations in forest regeneration areas (Natural Resources Institute Finland 2018). In most cases, the purpose of young stand management is to control competition between cultivated conifer species and naturally grown broadleaved trees. In addition, further costs arise from regular cuttings of brushwood along roads and railways as well as under electrical power lines. Mechanical cutting with a clearing saw is the most common method for sprout removal, but associated with vigorous re-sprouting (de Jong 2000; Wall 1990), and therefore, cutting operations should be repeated regularly, which increases management costs (Uotila 2017).

In order to avoid repeated cuttings, a considerable effort has been made to develop a biological sprout control method (Becker et al. 2005; Bellgard et al. 2014; Hamberg and Hantula 2016, 2018; Hamberg et al. 2014, 2015, 2017) for boreal and temperate vegetation zones where the biocontrol agent *Chondrostereum purpureum* (Pers. ex Fr.) Pouzar is common (Erikson and Ryvarden 1973; Ramsfield et al. 1999; Vartiamäki et al. 2008). *C. purpureum* is usually a saprophytic or parasitic pioneer species occurring especially on deciduous stumps, branches, and trunks. It may also cause silver leaf disease in fruit trees, but is able to infect them only through freshly wounded branches, stems, or roots (Becker et al. 2005; Butler and Jones 1949; de Jong 2000; Erikson and Ryvarden 1973; Gosselin et al. 1999; Hamberg et al. 2017). The fungus occurs sometimes also in conifers (Etheridge and Morin 1963; Ramsfield et al. 1996) but is not known to cause diseases in them (Gosselin et al. 1995).

As a biocontrol agent, *C. purpureum* is capable of preventing sprouting of cut stumps after it is spread as an inoculum (typically small segments of hypha diluted with water or gel-based product) on freshly cut stumps (Bellgard et al. 2014; Hamberg et al. 2015). Usually, within one to three growing seasons after the treatment, the fungus can penetrate towards roots (Hamberg et al. 2017), decay wood material, and finally kill a host tree (Hamberg and Hantula 2018; Wall 1986, 1991). This method is efficient in many broadleaved trees (Becker et al. 2005; Hamberg and Hantula 2016, 2018; Hamberg et al. 2015) but harmless for healthy nontarget trees (Gosselin et al. 1999). For example, in different birch species and alder, mortalities of treated stumps have been between 80 and 100% (Becker et al. 2005; Hamberg and Hantula 2018), and in European aspen ca. 80% (Hamberg and Hantula 2016).

Investigations on *C. purpureum* as a biocontrol agent have usually been performed in dry and sunny weather or during a very slight rain (Hamberg and Hantula 2016, 2018, 2020; Hamberg et al. 2015). Only minor effects due to such rains before and after the treatment have been observed (decrease and increase in birch stump mortality, respectively), but even if noticed, they have disappeared after two growing seasons (Hamberg and Hantula 2020; Vartiamäki et al. 2009). Thus, the outcome of possible rainstorms during the cutting and treatment of stumps is unknown. Such information is, however, obligatory for possible practical applications, since fungal inoculum is spread on freshly cut stumps as a water suspension (Hamberg and Hantula 2018), which may be prone to heavy rain washing away inoculum from the stump surface and reducing the efficacy of treatment if compared with one performed in sunny weather.

Earlier studies indicate that the performance of *C. purpureum* may be significantly enhanced by formulations improving fungal growth conditions (Harper et al. 1999; Lygis et al. 2012). One option would be to use adjuvants to increase the stability of *C. purpureum* inoculum on freshly cut stump surfaces. Adjuvants are formulation additives aimed to increase the effectiveness of the control agent (Calvo-Garrido et al. 2019; Larena et al. 2010): they may act by various mechanisms, such as enabling the spray to stick better by wetting the surface, preventing or accelerating the spray drying, increasing spray absorption, or enabling more uniform spreading of the spray over the surface (improved coverage) (Boyette 2006; Pacanoski 2015).

In this study, we tested a hypothesis stating that heavy rain would decrease the efficacy of *C. purpureum* treatment (i.e., mortality of broadleaved stumps). We also evaluated whether fixing the fungal inoculum on cut stump surfaces by adjuvants would increase the treatment efficacy since rain is one of the most detrimental issues for herbicide performance (Pacanoski 2015).

## Material and methods

### Field experiments

The experiment was performed in two medium-fertile forest regeneration areas of Norway spruce (*Picea abies* (L.) H. Karst.) in Loviisa, Southern Finland (60° 27′ 25″ N, 26° 13′ 30″ E). The first area was *Myrtillus* type forest (Cajander 1926) on coarse moraine soil, 4.7 ha in size and cut in 2012. The second one was *Oxalis-Myrtillus* type forest on fine moraine, 2.1 ha in size and cut in 2010. At both sites, broadleaved saplings were cut and soil prepared (mounding) in 2013, and spruce seedlings 1–2 years old were planted in 2014. By 2017, a dense cover of naturally regenerated birch (*Betula pendula* Roth and *Betula pubescens* Ehrh.) saplings had regrown at both sites.

Altogether, 640 birch (*B. pendula* and *B. pubescens*) saplings were included in the study (Table 1). *B. pendula* and *B. pubescens* were not investigated separately since their responses to the *C. purpureum* treatment do not differ significantly from each other (Hamberg et al. 2015). The saplings were marked with a tag and a plastic stick. Four different treatments were performed at both sites: (1) saplings were cut with a clearing saw and a suspension of *C. purpureum* inoculum strain R5 (Hamberg et al. 2015, DSMZ 28656) was applied on freshly cut stump surfaces using a plastic pressure sprayer (basal solution was provided by Verdera Ltd., Espoo, Finland, and it included 1.3 × 10^6^ colony forming units (CFU) per g, according to Gonzáles (1996), and was diluted 1:10 with tap water just before the treatment), (2) similar *C. purpureum* treatment but adjuvant Silwet Gold added (1 g per liter, i.e., 0.1% of the total weight of inoculum, Berner Ltd., Helsinki, Finland), (3) similar *C. purpureum* treatment with an adjuvant Polyhydra (4.5 g per liter, i.e., 0.5% of the total weight of inoculum, DF Green Ltd., Helsinki, Finland), and (4) the control (cutting only, no inoculum). All inoculums were spread on potato dextrose agar Petri plates before and after the treatments to verify their viability (all were viable).

**Table 1:** The number of investigated birch (*Betula pendula* and *Betula pubescens*) stumps and their diameter, height, and growth space (number of other saplings around) in different treatments where the fungus *Chondrostereum purpureum* was used as a control agent of sprouting, and fungus-free control

Condition	Treatment	Number of saplings	Amount of irrigation (mm m^−2^)^a^	Stump diameter (cm)	Stump height (cm)	Number of other saplings around^b^
Dry	*C. purpureum*	80	0	1.4 ± 0.6	17.5 ± 3.0	12.3 ± 5.2
	*C. purpureum* + Silwet Gold^c^	80	0	1.3 ± 0.5	17.5 ± 3.0	11.6 ± 4.9
	*C. purpureum* + Polyhydra^c^	80	0	1.2 ± 0.4	16.7 ± 3.0	12.8 ± 6.8
	Control	80	0	1.3 ± 0.5	19.4 ± 2.9	11.6 ± 4.7
Rainstorm	*C. purpureum*	80	24 ± 13	1.4 ± 0.5	18.2 ± 3.3	9.6 ± 3.4
	*C. purpureum* + Silwet Gold^c^	80	18 ± 11	1.4 ± 0.4	16.9 ± 3.0	9.1 ± 3.1
	*C. purpureum* + Polyhydra^c^	80	20 ± 11	1.3 ± 0.5	18.5 ± 4.2	8.4 ± 2.6
	Control	80	8 ± 2^d^	1.3 ± 0.5	20.6 ± 3.9	14.5 ± 4.4

Means ± standard deviations have been presented

^a^Measured using rain gauge, mean ± standard deviation per 0.5 h presented. The criteria for rainstorm is 5.5 mm m^−2^ per 0.5 h (Finnish Meteorological Institute 2017)

^b^Number of other saplings and stumps around an investigated stump within a radius of 0.5 m

^c^Adjuvant

^d^Windy weather moved water away from the control plots more than from the other treatment plots

Treatments in the field were performed on the 7th of June 2017. The weather was sunny (no rain) with a mean daily temperature of 16.2 °C (Natural Resources Institute Finland 2019; a description of the data set from Venäläinen et al. 2005). At each site, birch saplings were treated either under artificial rain (two clusters including all treatments) or with no rain (two clusters including all treatments). Each cluster, including all treatments, was irrigated from the middle of the cluster upwards by the Volunteer Fire Department of the municipality of Porlammi for 30 min: 15 min during the cutting and treatment of stumps and 15 min after that. The amount of irrigation was adjusted to correspond to a rainstorm, i.e., at least 5.5 mm m^−2^ per 0.5 h (Finnish Meteorological Institute 2017), and it was measured within each of the clusters treated under artificial rain using a rain gauge (model Tarmo, EAN: 6410412539328). In each irrigation cluster including all treatments, we started with a different treatment (in the first cluster with the first treatment, i.e., *C. purpureum* only; in the second cluster with the second treatment, etc.) and other treatments were performed in the same order as presented above. The same order of treatments was followed also in the other four blocks without artificial rain. A buffer zone of at least 4 m was used around the sample plots, and all of the saplings were cut between them. Stumps in treatments without artificial rain were covered by a plastic shelter that was supported by a wooden framework (forming a triangular tent open at both ends) for 5 days to ensure that natural rains did not affect the results. During these 5 days, i.e., 7th to 12th of June 2017, the mean maximum temperature was 19.8 °C (Natural Resources Institute Finland 2019; Venäläinen et al. 2005). We did not measure maximum temperature within the tents but based on earlier research, it may have been up to twice as high as the temperature outside the tents (Pinomaa 2016). During these 5 days when the tents were within the two regeneration areas, we measured the amount of rain using four rain gauges (model Tarmo, EAN: 6410412539328). Two rain gauges were located in each forest regeneration area, one next to a tent and the other located next to a cluster including sample plots treated under artificial rain. When the tents were removed on the 12th of June, it had rained 20–21 mm m^−2^ based on rain gauges (according to weather records 29 mm m^−2^, Natural Resources Institute Finland 2019; Venäläinen et al. 2005), and all shelters were intact, i.e., with no leakage of rainwater.

Saplings were investigated in September 2017, 2018, and 2019. In 2017, one growing season after the treatments (3 months), we measured the diameter (mm) and the height of the stumps (from the base to the cut surface, cm), and counted the number of other saplings and stumps around an investigated stump (*r* = 0.5 m). Furthermore, in 2017, 2018, and 2019, we counted the number of living stump sprouts, measured the height of the tallest stump sprout per stump (cm), and counted the number of fruiting bodies of *C. purpureum* per stump (as a class: 0 = no fruiting bodies, 1 = 1–3 fruiting bodies, 2 = 4–10 fruiting bodies, 3 = more than 10 fruiting bodies). Traces of hare or moose browsing were also recorded (0 = no browsing, 1 = browsing observed).

Wood samples were randomly taken from 160 stumps (5 per sample plot) on October 16–17 in 2017 to investigate whether *C. purpureum* had penetrated to treated stumps. On these days, weather was cloudy with a temperature of ca. 9 °C and very slight rain (ca. 3 mm, Natural Resources Institute Finland 2019; Venäläinen et al. 2005). All equipment, knife, drill bit, and cut plastic bottle (collector) for collecting wood chips, were sterilized with diethyl ether (VWR International Ltd., Helsinki, Finland) before sampling from each stump. First, bark was removed from the upper parts of stumps (from one side), ca. 1–5 cm from the cut surface downwards, with a sterilized knife, and the fresh wood was surface sterilized with ether. Second, samples were drilled from the sterilized area with a sterilized 4–5 mm drill bit; wood chips (ca. 0.28 g per sample) were collected in a sterilized plastic container and moved to small labelled paper bags, with one sample per bag. Third, the holes created by drilling were covered with a protective agent (Cooper Bio Haavasuoja, Berner Ltd., Helsinki, Finland) to improve wound recovery from drilling in order to minimize penetration of other pathogens.

### Investigations in the laboratory

Ca. 0.02 g of fresh wood chips were extracted for fungal DNA from stump samples (Nucleo Spin® Soil, Macherey-Nagel Inc., Düren, Germany) in order to verify the presence of *C. purpureum* strain R5 in treated stumps. Master mix, 25 μl, was prepared as follows: 20.4 μl DDW, 2.5 μl Dream Taq™ buffer (Thermo Scientific™, Vantaa, Finland), 0.5 μl dNTP (10 μM), 0.5 μl forward primer (25 μM), 0.5 μl reverse primer (25 μM), 0.1 μl DreamTaq DNA polymerase (Thermo Scientific™, Vantaa, Finland), and 0.5 μl of DNA sample. Four pairs of primers with increased specificity to *C. purpureum* strain R5 were used in PCR analyses (Hamberg et al. 2017). To verify the PCR result, ca. 11% of the samples were investigated again, starting from wood chips. For the rest of the samples, only PCR was performed twice, and in the cases that the amplification was inefficient (only a faint band in a gel) in the first round, the amount of template was doubled.

Furthermore, three wood chips were placed on water agar in order to cultivate the inoculated *C. purpureum* strain R5 from them. In case a hypha of *C. purpureum* was growing out of the chip, a single hyphal tip was moved onto Potato Dextrose agar (PDA: 24 g potato dextrose broth and 15 g agar with 1000 ml deionized water; Becton, Dickinson and Company, Franklin Lakes, NJ, USA). After hyphal growth, a piece from the mycelial edge was moved onto PDA agar covered with cellophane agar. After the hypha was grown on a cellophane PDA, a piece of ca. 2 mm^2^ was moved to a tube for DNA extraction (Vainio et al. 1998). The success of DNA extraction was verified by PCR with a RAMS (Random Amplified Microsatellites) or a minisatellite M13 primer as described by Hamberg et al. (2017): 19.4 μl DDW, 2.5 μl DreamTaq buffer, 0.5 μl dNTP, 2 μl primer, 0.1 μl DreamTaq polymerase, and 0.5 μl DNA sample. DNA concentration was determined with NanoDrop, and samples were diluted 1:10 when the concentration was less than 10 ng μl^−1^, 1:20 when the concentration was 10 ≤ × < 100 ng μl^−1^, and 1:100 when it was ≥ 100 ng μl^−1^.

*C. purpureum* strain R5 specific primers were used to test whether the hyphae originated from the strain R5. Master mix (AllTaq Master Mix Kit, Qiagen Group, Helsinki, Finland) was used in 20 μl PCR reactions as follows: 5 μl Master Mix (including enzyme, buffer, and dNTP), 14.5 μl DDW, 0.2 μl forward primer (25 μM), 0.2 μl reverse primer (25 μM), and 0.1 μl DNA sample. PCR conditions were modified from Hamberg et al. (2017): (1) 95 °C for 2 min, (2) 95 °C for 5 s, (3) 57/59 °C for 15 s, (4) 72 °C for 10 s, and (5) cooling to 4 °C. Phases 2–4 were repeated 36 times for the primer pairs R5CGA800F/R and R5CCA1200F/R, 38 times for R5GT600F/R17, and 40 times for R5GTG390F/RUA. All reactions were done twice (5% starting from the DNA extraction), and in case a marker was not visible, PCR was performed again with a dilution of 1:10, 1:20, or 1:100. The hypha was considered the *C. purpureum* strain R5 if amplifications with all four R5 specific primer pairs resulted in amplification products.

### Statistical analyses

Effects of the treatment conditions (dry weather and artificial rainstorm) on the mortality of birch stumps (i.e., no living stump sprouts) were investigated with generalized linear mixed models (GLMMs) in the statistical program R, in library *lme4*, using function *glmer*, and binomial distribution with logit link (Bates et al. 2015; R Core Team 2018). All stumps were included in these models. The effects on the number of stump sprouts per stump were investigated using *glmer* function assuming Poisson distribution with log link, and on the maximum height of stump sprouts assuming normal distribution (with identity link) in library *nlme* using function *lme* after the response was log transformed (Pinheiro et al. 2018). In stump sprout models (number and maximum height), only living stumps, having at least one sprout, were included. Data for each treatment, i.e., for (1) *C. purpureum*, (2) *C. purpureum* with an adjuvant Silwet Gold, (3) *C. purpureum* with an adjuvant Polyhydra, and (4) control, were analyzed separately.

Each model included explanatory variables as follows: (1) interaction between condition (a factor with two levels: dry weather or artificial rainstorm) and year (a factor with three levels: 2017, 2018, and 2019), (2) diameter of a stump (mm), (3) height of a stump (cm), (4) the number of other stumps and saplings around an investigated stump, and (5) a factor with two levels indicating whether a wood sample had been taken from a stump (0 = not drilled, 1 = wood sample drilled from a stump). Furthermore, a factor indicating whether hare or moose browsing was visible (0 = no browsing, 1 = browsing observed) was added to the maximum height of stump sprout models. Explanatory variables 2–4 (and browsing for the maximum height models) were included in the models because these may have an effect on the mortality, on the number of, or on the maximum height of stump sprouts (Hamberg et al. 2015). Furthermore, because drilling may increase mortality and affect the ability of a stump to produce stump sprouts, we separated it from the effects of the treatment condition (dry vs. rainstorm). Site and cluster (including four different treatments) were included in the models as nested random factors since stumps and sample plots within a site may be more similar than randomly collected birch stumps. All figures in the “Results” section have been drawn using R and are presented using predictions based on the models (R Core Team 2018).

## Results

The DNA of the *C. purpureum* strain R5 was frequently amplified with direct amplification from the wood of the treated birch (*B. pendula* and *B. pubescens*) stumps but never from the control stumps (Table 2). The frequencies were relatively similar on stumps subjected to rainy and dry conditions. *C. purpureum* grew also from wood chips drilled from stumps that were infected with the fungal strain R5, 83 times, i.e., ca. 52% of the wood samples collected (*n* = 160), verifying Koch’s postulates. Almost all fungal strains grown from wooden samples were proven to be *C. purpureum* strain R5 used for inoculations, showing that the fungus had successfully penetrated the stumps, both cut and treated (Table 2). Only in a few cases, the detected *C. purpureum* was another strain than R5. Fungal strains grew also from two control stumps, but none of them was identified as strain R5.

**Table 2:** Frequency of the decay fungus *Chondrostereum purpureum*, strain R5, used in the study, on birch, *Betula pendula* and *Betula pubescens*, stumps ca. 4 months after the treatments

Condition	Treatment	Frequency (%)			
		*n*	Directly from wood samples	*n*	From fungal hypha grown from wooden chips
Dry	*C. purpureum*	20	100	14	100
	*C. purpureum* + Silwet Gold^a^	20	90 ± 7	16	88 ± 9
	*C. purpureum* + Polyhydra^a^	20	100	12	100
	Control	20	0	0	-
Rainstorm	*C. purpureum*	20	100	11	100
	*C. purpureum* + Silwet Gold^a^	20	85 ± 8	14	100
	*C. purpureum* + Polyhydra^a^	20	75 ± 10	14	93 ± 7
	Control	20	0	2	0

The occurrence was verified by PCR using R5 specific primers (see Hamberg et al. 2017). Means ± standard errors have been presented

^a^Adjuvant

**Fig. 1:**
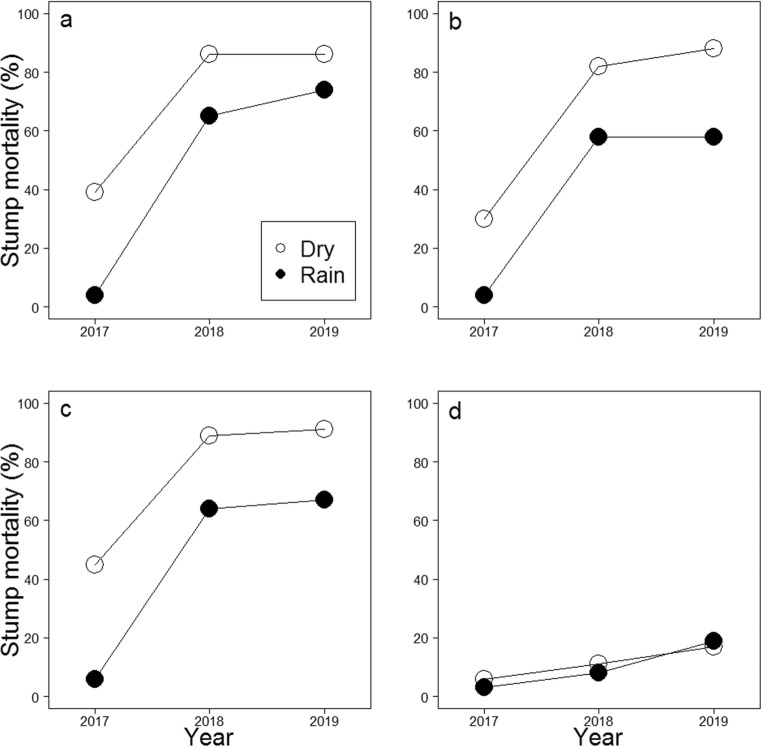
Predicted values for birch (*Betula pendula* and *Betula pubescens*) stump mortality based on the GLMMs (Table 3) for **a** the *Chondrostereum purpureum* treatment (*n* = 480), **b** the *C. purpureum* with the adjuvant Silwet Gold treatment (SG, *n* = 480), **c** the *C. purpureum* with the adjuvant Polyhydra treatment (PH, *n* = 480), and for **d** the control (no *C. purpureum* application on stump surfaces, *n* = 480) when the treatments have been performed under artificial rainstorm (rain) or without it (dry). Predictions for one (2017), two (2018), and three (2019) growing seasons after the treatments have been counted using mean values for stump diameter, stump height, and other saplings and stumps around an investigated stump, and assuming no drilling

One growing season after the treatments (i.e., after 3 months), mortality of birch stumps was lower in stumps treated with *C. purpureum* under artificial rain compared with stumps treated in dry conditions (*p* ≤ 0.001, Table 3, Fig. 1). However, in the pure *C. purpureum* treatment without adjuvants, the mortality of birch stumps treated under artificial rain increased considerably quicker (from 4% in 2017 to 74% in 2019) than in dry condition (from 39 to 86%, *p* < 0.004) so that by the end of the experiment (2019) there was no statistically significant difference between treatments conducted under artificial rain and in dry conditions. In the *C. purpureum* treatments with adjuvants (both Silwet Gold and Polyhydra) performed under artificial rain, the mortality increased from 4–6% up to 58–67% in 2017–2019 and stayed at lower levels than in the treatments performed in dry weather (increase from 30–45% up to 88–91% in years 2017–2019, the curves for dry and artificial rain did not differ, *p* ≥ 0.177, see Table 3, Fig. 1). In the control (cutting only, no *C. purpureum* application on birch stumps), stump mortality increased from the first inventory in 2017 to the last in 2019 similarly both in artificial rain (from 3 to 19%) and dry conditions (6 to 17%, *p* ≥ 0.289).

**Table 3:** The effect of artificial rainstorm during the *Chondrostereum purpureum* treatment on the mortality of birch (*Betula pendula* and *Betula pubescens*) stumps, and the number and maximum height of stump sprouts (generalized linear mixed models, GLMMs)

Model Treatment^a^	*n*	Intercept Coeff. ± SE	*p*	Rainstorm^b^ Coeff. ± SE	*p*	Year 2018^c^ Coeff. ± SE	*p*	Year 2019^c^ Coeff. ± SE	*p*	Rainstorm: Year 2018^d^ Coeff. ± SE	*p*	Rainstorm: Year 2019^d^ Coeff. ± SE	*p*
Stump mortality													
*C. purp*	480	**−***3.074* ± *1.084*	*0.005*	**−***2.849* ± *0.685*	*< 0.001*	*2.282* ± *0.414*	**<***0.001*	*2.282* ± *0.414*	**<***0.001*	*1.642* ± *0.713*	*0.021*	*2.061* ± *0.723*	*0.004*
*C. purp* SG	480	**−***3.371* ± *0.940*	**<***0.001*	**−***2.312* ± *0.592*	**<***0.001*	*2.400* ± *0.393*	**<***0.001*	*2.851* ± *0.423*	**<***0.001*	1.092 ± 0.691	0.114	0.640 ± 0.708	0.365
*C. purp* PH	480	**−***4.115* ± *1.007*	**<***0.001*	**−***2.530* ± *0.731*	*0.001*	*2.314* ± *0.433*	**<***0.001*	*2.525* ± *0.449*	**<***0.001*	0.283 ± 0.657	0.133	0.902 ± 0.669	0.177
Control	480	− 0.535 ± 1.351	0.692	− 0.787 ± 0.785	0.317	0.677 ± 0.593	0.254	*1.136* ± *0.565*	*0.044*	0.456 ± 0.931	0.624	0.934 ± 0.881	0.289
Number of stump sprouts													
*C. purp*	208	*1.465* ± *0.325*	**<***0.001*	− 0.062 ± 0.119	0.601	**−***0.746* ± *0.209*	**<***0.001*	**−***0.951* ± *0.228*	**<***0.001*	0.300 ± 0.248	0.226	0.278 ± 0.278	0.318
*C. purp* SG	229	*1.468* ± *0.307*	**<***0.001*	− 0.207 ± 0.179	0.249	**−***0.658* ± *0.171*	**<***0.001*	**−***1.062* ± *0.234*	**<***0.001*	*0.473* ± *0.205*	*0.021*	0.490 ± 0.268	0.068
*C. purp* PH	205	*1.484* ± *0.342*	**<***0.001*	0.048 ± 0.246	0.845	**−***0.722* ± *0.217*	*0.001*	**−***0.977* ± *0.256*	**<***0.001*	0.383 ± 0.251	0.126	0.456 ± 0.290	0.117
Control	426	*0.844* ± *0.203*	**<***0.001*	− 0.016 ± 0.088	0.860	**−***0.316* ± *0.085*	**<***0.001*	**−***0.727* ± *0.099*	**<***0.001*	− 0.035 ± 0.120	0.771	0.093 ± 0.138	0.501
Maximum height of stump sprouts ^e^													
*C. purp*	208	*3.648* ± *0.233*	**<***0.001*	0.002 ± 0.122	0.987	*0.632* ± *0.101*	**<***0.001*	*0.834* ± *0.101*	**<***0.001*	− 0.203 ± 0.125	0.107	− 0.087 ± 0.129	0.499
*C. purp* SG	229	*3.351* ± *0.178*	**<***0.001*	0.024 ± 0.107	0.835	*0.609* ± *0.086*	**<***0.001*	*0.821* ± *0.099*	**<***0.001*	− 0.026 ± 0.107	0.812	0.037 ± 0.118	0.756
*C. purp* PH	205	*3.262* ± *0.202*	**<***0.001*	0.199 ± 0.165	0.281	*0.494* ± *0.103*	**<***0.001*	*0.760* ± *0.109*	**<***0.001*	0.058 ± 0.126	0.379	0.008 ± 0.133	0.953
Control	426	*3.432* ± *0.145*	**<***0.001*	− 0.030 ± 0.115	0.802	*0.589* ± *0.054*	**<***0.001*	*0.900* ± *0.055*	**<***0.001*	0.064 ± 0.076	0.402	*0.163* ± *0.077*	*0.035*

Coefficients with standard errors (Coeff. ± SE) have been presented. Statistically significant results have been indicated in italic. Living stumps only have been included in the models concerning stump sprouts (number and maximum height). The effects of stump diameter and height, the number of other sapling and stumps around an investigated sapling, and drilling and browsing on the responses have been explained in text

^a^*C. purp* is *C. purpureum* treatment without adjuvants, i.e., saplings were cut and a fungal inoculum including *C. purpureum* as an sprout control agent was applied on freshly cut stumps; *C. purp* SG is *C. purpureum* treatment with an adjuvant Silwet Gold; *C. purp* PH is *C. purpureum* treatment with an adjuvant Polyhydra; and in Control, saplings were cut only (no *C. purpureum* application)

^b^Difference between dry weather and artificial rain (rainstorm) in 2017

^c^Difference between year 2017, and 2018 and 2019 in dry weather

^d^Difference between the curves in dry and artificial rain (rainstorm) weather in 2018 and 2019

^e^The response was log transformed

**Fig. 2:**
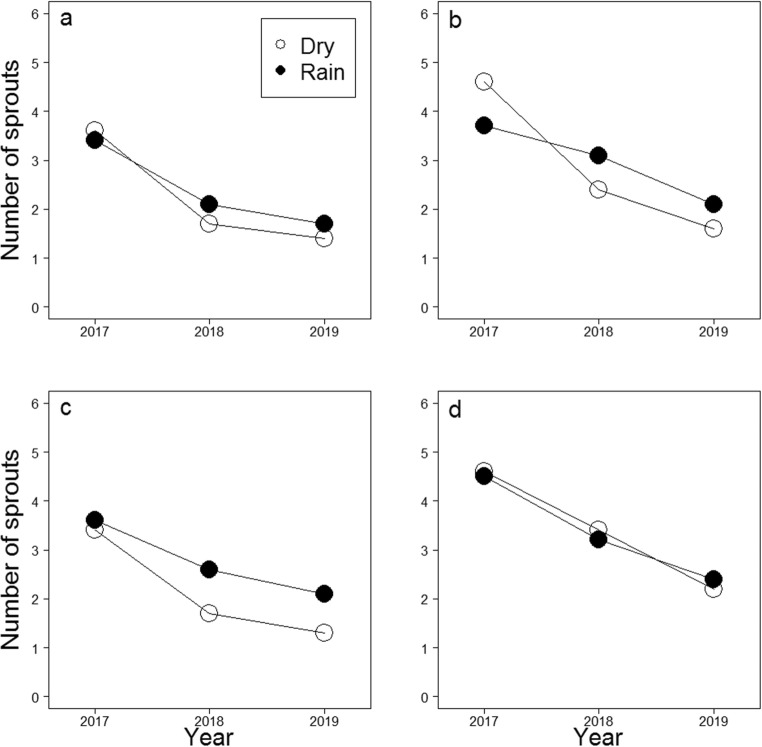
Predicted values for the number of birch (*Betula pendula* and *Betula pubescens*) stump sprouts per living stump based on the GLMMs (Table 3) for **a** the *Chondrostereum purpureum* treatment (*n* = 208), **b** the *C. purpureum* with the adjuvant Silwet Gold treatment (SG, *n* = 229), **c** the *C. purpureum* with the adjuvant Polyhydra treatment (PH, *n* = 205), and for **d** the control (no *C. purpureum* application on stump surfaces, *n* = 426) when the treatments have been performed under artificial rainstorm (rain) or without it (dry). Predictions for one (2017), two (2018), and three (2019) growing seasons after the treatments have been counted using mean values for stump diameter, stump height, and other saplings and stumps around an investigated stump, and assuming no drilling

The number of stump sprouts per living stump decreased from the first inventory in 2017 to the last inventory in 2019 similarly both in rainstorm and dry conditions in all treatments and the control (*p* ≥ 0.068), except in the *C. purpureum* treatment with the adjuvant Silwet Gold (*p* = 0.021, Table 3, Fig. 2). In this treatment, the number of stump sprouts per stump decreased more slowly in stumps treated under artificial rain than in stumps treated without artificial rain.

**Fig. 3:**
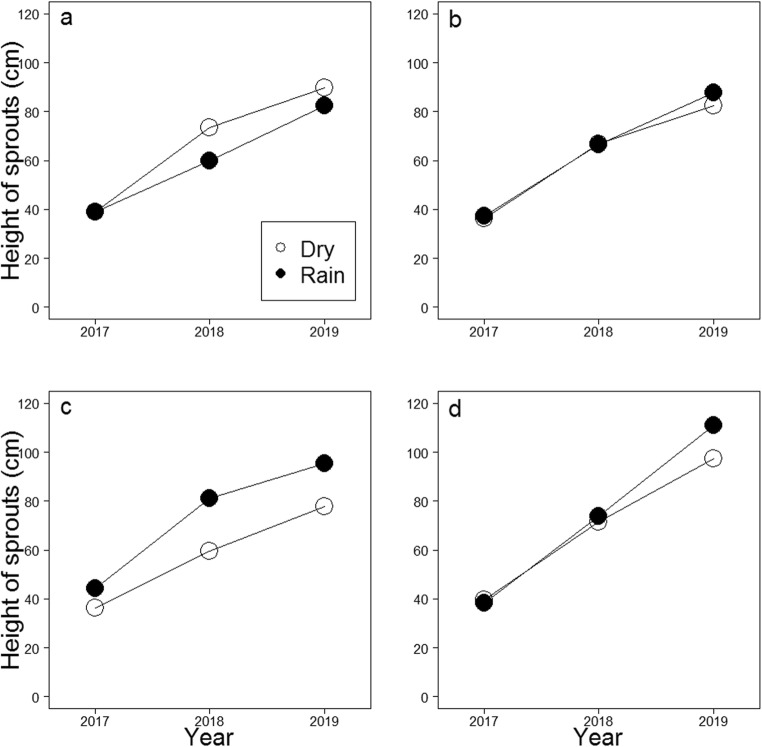
Predicted values for the maximum height of birch (*Betula pendula* and *Betula pubescens*) stump sprouts based on the GLMMs (Table 3) for **a** the *Chondrostereum purpureum* treatment (*n* = 208), **b** the *C. purpureum* with the adjuvant Silwet Gold treatment SG, *n* = 229), **c** the *C. purpureum* with the adjuvant Polyhydra treatment (PH, *n* = 205), and for **d** the control (no *C. purpureum* application on stump surfaces, *n* = 426) when the treatments have been performed under artificial rainstorm (rain) or without it (dry). Predictions one (2017), two (2018), and three (2019) growing seasons after the treatments have been counted using mean values for stump diameter, stump height, and other saplings and stumps around an investigated stump, and assuming no drilling and browsing

In all *C. purpureum* treatments, the maximum height of stump sprouts increased from the first inventory in 2017 to the last inventory in 2019 similarly in stumps treated under artificial rain and in dry conditions (*p* ≥ 0.107, Table 3, Fig. 3). However, in the control treatment without *C. purpureum*, sprouts grew faster after cutting under artificial rain (*p* = 0.035).

Larger stumps (in terms of stump diameter) died faster than smaller stumps in all *C. purpureum* treatments (GLMMs, *p* ≤ 0.001 for all). However, in the control treatment (cutting only), living larger stumps yielded more sprouts (*p* < 0.001), and in the *C. purpureum* treatment with the adjuvant Silwet Gold and the control, they yielded taller stump sprouts (*p* = 0.050 and *p* = 0.007, respectively) than smaller stumps. In the control, stump mortality was lower (*p* = 0.003) and the number of stump sprouts per stump was higher (*p* = 0.042) in taller than shorter stumps. In the *C. purpureum* treatment with the adjuvant Polyhydra, the number of stump sprouts decreased with increasing number of other saplings and stumps around an investigated stump (*p* = 0.050). Drilling did not affect stump mortality. In the *C. purpureum* treatment with the adjuvant Silwet Gold, the maximum height of stump sprouts was higher in drilled than in non-drilled stumps (*p* = 0.024) but in the *C. purpureum* treatment with the adjuvant Polyhydra, the maximum height of stump sprouts was lower in drilled than in non-drilled stumps (*p* = 0.035). Browsing did not affect the maximum height of birch stump sprouts in different treatments.

In *C. purpureum* treatments, fruiting bodies were most frequent after the second growing season (Table 4). It seems that development of fruiting bodies was slightly delayed after treatments performed under artificial rainstorms, since after the third growing season, they were more abundant than in stumps treated in the dry condition. In the control (cutting only), the occurrence of fruiting bodies was low.

**Table 4:** Occurrence of fruiting bodies of the decay fungus *Chondrostereum purpureum* on birch, *Betula pendula* and *Betula pubescens*, stumps in the different treatments and conditions one, two, and three growing seasons after the treatments in 2017, 2018, and 2019

Condition	Treatment	Occurrence (%)					
		*n*	After the first growing season	*n*	After the second growing season	*n*	After the third growing season
Dry	*C. purpureum*	80	16 ± 4	80	50 ± 6	80	0
	*C. purpureum* + Silwet Gold^a^	80	9 ± 3	80	56 ± 6	80	4 ± 2
	*C. purpureum* + Polyhydra^a^	80	15 ± 4	80	58 ± 6	80	0
	Control	80	0	80	1 ± 1	80	0
Rainstorm	*C. purpureum*	80	8 ± 3	80	60 ± 6	80	10 ± 3
	*C. purpureum* + Silwet Gold^a^	80	4 ± 2	80	51 ± 6	80	4 ± 2
	*C. purpureum* + Polyhydra^a^	80	6 ± 3	80	54 ± 6	80	8 ± 3
	Control	80	0	80	4 ± 2	80	1 ± 1

Means ± standard errors have been presented

^a^Adjuvant

## Discussion

Our results revealed that heavy rain caused delays in the efficacy of the *C. purpureum* treatment against sprouting of birch (*B. pendula* and *B. pubescens*). However, in the pure *C. purpureum* treatment, this effect was only temporary since three growing seasons after the treatment there was no significant difference in stump mortalities when stumps were treated under heavy irrigation or without it. This agrees with previous studies, where natural (slight) rain has caused only temporal (Hamberg and Hantula 2020) or insignificant effects (Vartiamäki et al. 2009) on the efficacy of *C. purpureum* in preventing stump sprouting. These minor effects have possibly been due to a better moisture level for *C. purpureum* to grow (Boddy 1983a, b), but the delay observed here was probably caused by a rinsing of fungal mycelium from the stump surface under the heavy irrigation. This was also supported by the fact that in the treatments under heavy rain, fruiting bodies still occurred three growing seasons after the treatments, unlike in the treatments performed in dry conditions: fruiting bodies are known to occur for a longer time in trees that die slowly (Wall 1997). Thus, in possible practical applications, the number of colony-forming units should be high enough in fungal inoculum so that at least some hyphal fragments will stay on stump surfaces after heavy rain until they have grown into the wood. No clear differences between the three different *C. purpureum* treatments were found in dry weather, as all *C. purpureum* treatments yielded more than 80% mortalities corresponding to earlier results with birch (*B. pendula* and *B. pubescens*) saplings (Hamberg and Hantula 2018; Hamberg et al. 2015; Vartiamäki et al. 2009).

Unfortunately, adjuvants which aimed to help the spray adhere better seemed inefficient, since mortalities in the treatments under artificial rain remained at a lower level than in the treatments in dry conditions. This was disappointing as in fruit plants, the use of adjuvants has been proven to improve cell adherence and persistence of biological control agents on plant tissues (Calvo-Garrido et al. 2019; Larena et al. 2010). Similarly, adjuvants also greatly improved the biological control potential of the fungus, *Colletotrichum gloeosporioides* (Penz.) Penz & Sacc. against sicklepod, *Senna obtusifolia* (L.) Irwin & Barnaby, which is a serious weed pest in the southeastern US (Boyette 2006). The adjuvants tested in the present study should be further developed before their application to increase the efficacy of biological sprout control under rainy conditions can be considered.

The number and maximum height of stump sprouts did not differ between the *C. purpureum* treatments, but the general feature was that the number of stump sprouts decreased whereas the maximum height of stump sprouts increased from year 2017 to 2019 (see also Laine et al. 2019a). In the control, it seemed that the number and maximum height of stump sprouts was slightly higher—but not significantly different—than in the fungal treatments, which agrees with the results of previous research (Hamberg et al. 2015).

In this study, stump mortality was higher in stumps with larger diameters than in smaller stumps (similarly as in Hamberg et al. 2014; Laine et al. 2019b). Possibly, the larger stump surface enables fungal fragments to reach it more easily, and definitely, there is more space for a higher number of fungal fragments to colonize it. In large living stumps, the number and maximum height of stump sprouts was higher than in smaller stumps, possibly due to the better resources in the larger root systems of these plants (Aosaar et al. 2013; Laine et al. 2019a, b). Similarly, increase in competition (more saplings and stumps) around an investigated sapling was associated with lower number of stump sprouts indicating that limited resources within an area decreases the possibilities for the recovery of single plants (see also Atkinson 1992; Hamberg and Hantula 2018; Jones and Harper 1987; Laine et al. 2019a, b). Furthermore, lower stump mortality and higher sprout number per stump was associated with taller stumps. This highlights the fact that it is important not to leave tall stumps during cutting.

In our experiment, diethyl ether was used to sterilize drill bits and other equipment between the samplings. Based on our observations and results, it seemed that this method is relatively good: no cross-contamination was observed between the control and the *C. purpureum* treatments since *C. purpureum* hyphae grew only from two control stumps but none of the hyphae belonged to the applied fungal strain R5. Furthermore, ether is relatively easy to use in the field since it evaporates quickly so that there is no need for long waiting times between different samples. Yet, more research is needed to verify that ether can be used in sampling of environmental DNA, where cross-contamination may occur even from dead tissues, although in this, study direct DNA extraction from wood chips yielded highly similar results as the more time-consuming cultivation of fungal hyphae from wood chips followed by isolations of single hyphae and DNA extraction from a mycelial culture.
